# Peripheral vascular disease assessment in the lower limb: a review of current and emerging non-invasive diagnostic methods

**DOI:** 10.1186/s12938-018-0494-4

**Published:** 2018-05-11

**Authors:** Elham Shabani Varaki, Gaetano D. Gargiulo, Stefania Penkala, Paul P. Breen

**Affiliations:** 1 The MARCS Institute for Brain, Behaviour & Development, Western Sydney University, Penrith, NSW 2750 Australia; 2 School of Science and Health, Western Sydney University, Penrith, NSW 2750 Australia; 3 Translational Health Research Institute, Western Sydney University, Penrith, NSW 2750 Australia

**Keywords:** Peripheral arterial disease, Chronic venous insufficiency, Deep vein thrombosis, Plethysmography, Doppler ultrasound, Ankle Brachial Index

## Abstract

**Background:**

Worldwide, at least 200 million people are affected by peripheral vascular diseases (PVDs), including peripheral arterial disease (PAD), chronic venous insufficiency (CVI) and deep vein thrombosis (DVT). The high prevalence and serious consequences of PVDs have led to the development of several diagnostic tools and clinical guidelines to assist timely diagnosis and patient management. Given the increasing number of diagnostic methods available, a comprehensive review of available technologies is timely in order to understand their limitations and direct future development effort.

**Main body:**

This paper reviews the available diagnostic methods for PAD, CVI, and DVT with a focus on non-invasive modalities. Each method is critically evaluated in terms of sensitivity, specificity, accuracy, ease of use, procedure time duration, and training requirements where applicable.

**Conclusion:**

This review emphasizes the limitations of existing methods, highlighting a latent need for the development of new non-invasive, efficient diagnostic methods. Some newly emerging technologies are identified, in particular wearable sensors, which demonstrate considerable potential to address the need for simple, cost-effective, accurate and timely diagnosis of PVDs.

**Electronic supplementary material:**

The online version of this article (10.1186/s12938-018-0494-4) contains supplementary material, which is available to authorized users.

## Background

Peripheral vascular disease (PVD) is a major cause of morbidity and mortality globally, with significant financial burdens on critical healthcare resources [1–10]. Vascular diseases result from circulatory system dysfunction caused by damage, occlusion and/or inflammation of arteries and/or veins [11]. Peripheral arterial disease (PAD), chronic venous disease (CVD), which includes chronic venous insufficiency (CVI) and deep vein thrombosis (DVT), are common types of PVDs and are the most prevalent in the lower extremities.

Peripheral arterial disease (PAD) occurs with narrowing or blockage of the arteries with the formation of fatty deposits/plaque also known as atheroma. The body segment supplied by the impaired artery is then deprived of oxygen-rich blood and nutrients [12], often resulting in pain and numbness [13]. PAD can increase the risk of infection in the affected area, with severe occlusion increasing the risk of gangrene and ultimately amputation [14, 15]. PAD also increases the risk of coronary heart disease and cerebral vascular disease [2, 13]. In general, PAD affects 10–15% of the population and about 20% of people aged over 60 years [2, 16]. Worldwide, the incidence of PAD has increased from 164 million in 2000 to 202 million in 2010 [17].

Chronic venous insufficiency (CVI), also known as post thrombotic syndrome, occurs with unrestrained ambulatory venous hypertension associated with venous wall and value incompetence [18, 19]. Normal venous return occurs against gravity when the limb in is dependency (i.e. standing) and is achieved through active compression of the lower limb veins via contraction of muscles of the feet and legs [20]. Retrograde flow is prevented by a system of one-way valves in the veins [20]. However, extended periods of sitting or standing can lead to pooling of blood and an increase in venous hypertension in the lower limb. Although leg veins are usually able to tolerate increased venous pressure over short periods of time, extended periods of increased venous pressure can lead to stretched vein walls and damaged venous valves, ultimately leading to CVI [20, 21]. CVI symptoms range from a vague feeling of heaviness in lower extremities, swelling of the legs, aching, itching, skin colour changes and venous ulceration, particularly of the medial ankle [20]. The overall prognosis of venous ulceration is poor, with poor skin nutrition, delayed healing and recurrent ulceration [7, 8]. More than half of venous ulcerations require extensive therapy lasting more than 1 year [8, 22]. Disability caused by venous ulceration leads to loss of productive work hours (estimated at 2 million workdays/year) and early retirement [8, 23]. In addition, the financial burden of venous ulceration to healthcare systems in western countries is reported to be more than $3 billion annually [3, 8].

Deep vein thrombosis occurs with the formation of a blood clot in the deep venous system, most commonly in the lower limbs (i.e. superficial femoral and popliteal veins in the thighs and the posterior tibial and peroneal veins in the calves) [24]. While some patients with a DVT report leg pain, swelling, tenderness and redness of the affected area, not all are symptomatic which can delay life preserving diagnosis [24–26]. The most serious consequence of a DVT is a pulmonary embolism, which occurs when part of the thrombus dislodges and deposits in the vessels of the lung occluding circulation, ultimately leading to disability or death [24, 25]. Population studies have estimated an annual incidence of DVT of 0.5–1 per 1000 in the general population [6, 24–26]. Approximately one-third of patients with DVT develop a pulmonary embolism of whom about 20% of patients die before diagnosis or on the 1st day of diagnosis [25, 27].

Early diagnosis and management of PVD is crucial to address the high rates of mortality and morbidity, however around 50% of people are asymptomatic and therefore do not necessary seek medical assistance, or are not screened by clinicians in the absence of diagnosed disease. Establishing effective and efficient clinical non-invasive diagnostic tools to determine vascular competence is essential particularly for asymptomatic PVD patients who have the same risk of morbidity and mortality as those with more obvious symptoms [2, 28, 29]. A variety of both invasive and non-invasive diagnostic devices have been developed since the 1670s to facilitate accurate diagnosis and address the prevalence and socioeconomic impacts of PVDs [30, 31]. Four primary invasive methods are angiography, venography, ambulatory venous pressure, and intravascular ultrasound [32]. Although these invasive methods are highly accurate, they are expensive, uncomfortable for patients and carry inherent risks. These methods are typically reserved for instances where highly detailed measurements are required, for example, before surgical planning, during intervention procedure, and or in virtual surgery systems [32–34]. However, the level of risk and discomfort is not appropriate for routine screening, but which is crucial for early disease diagnosis when preventative care could have the greatest benefit. Non-invasive methods are an alternative and can be used more routinely for diagnosis and follow-up of subsequent treatment. However, each method has limitations, which continue to motivate the development of new diagnostic and clinically applied methods.

This paper provides a comprehensive review of the available non-invasive methods for the assessment of peripheral hemodynamic function in the lower extremities and recommendations for an ideal non-invasive diagnostic tool to confirm/exclude the presence of PVDs. Three primary validated non-invasive technologies (plethysmography, Doppler ultrasound and blood pressure methods) are discussed in detail. Subsequently, emerging diagnostic techniques are presented. The limitations and strengths of each method are identified, followed with recommendations for an ideal non-invasive method to diagnose PVDs.

## Plethysmographic methods

**Fig. 1:**
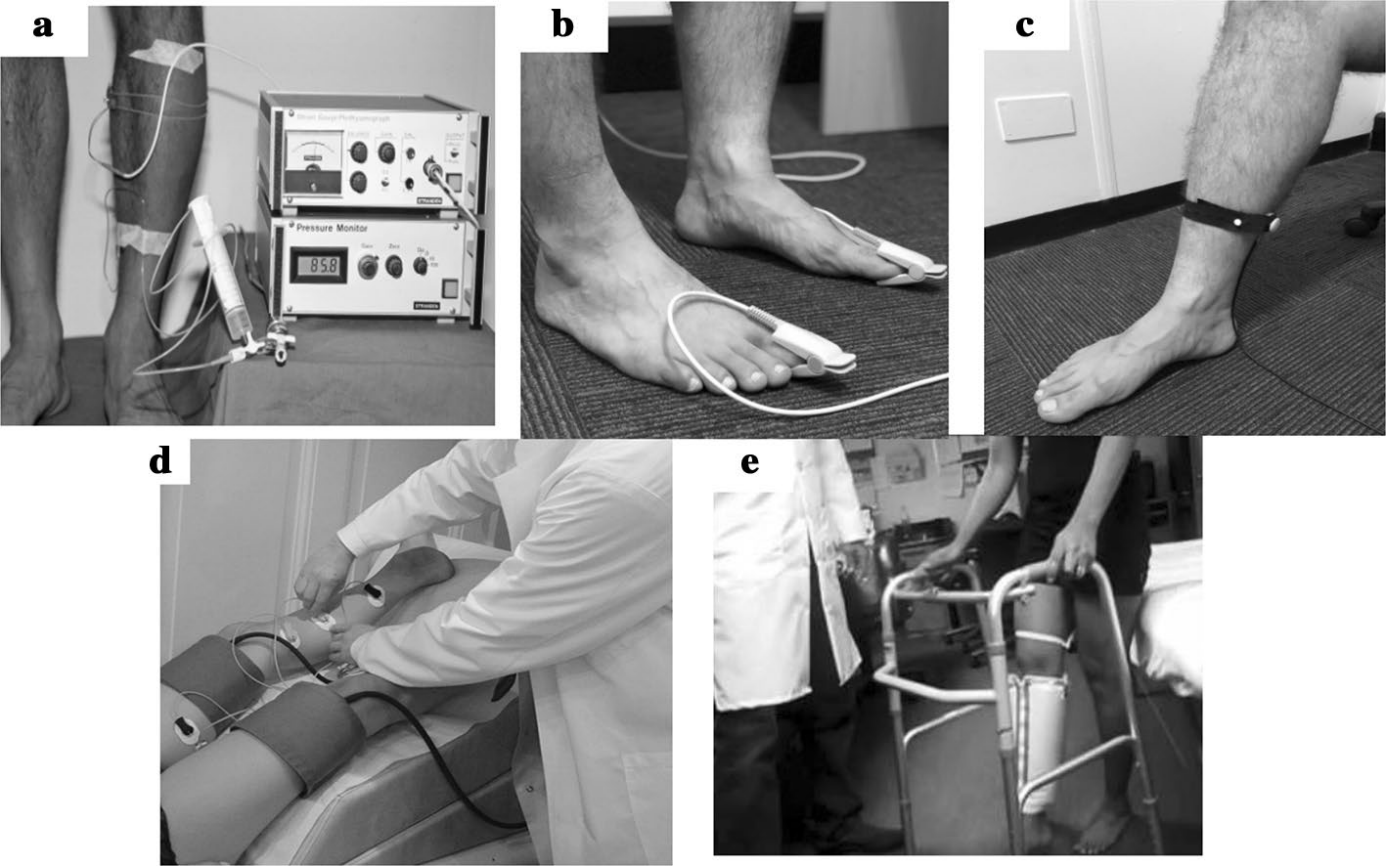
Examples of peripheral vascular function assessment in the lower limb using plethysmography techniques; **a** strain gauge plethysmography [37]; **b** photo plethysmography (PPG); **c** quantitative PPG/light reflection rheography (LRR); **d** Impedance Plethysmography, modified from [38]; **e** Air Plethysmography (APG) [39]

Plethysmography measures blood volume changes in the lower extremities. The principle of plethysmography for limb volume measurement was first introduced by Francis Glissonioin in 1677 using water displacement [30, 31]. Later, the classic water plethysmography method was modified to other measures of volume (i.e. strain gauge, photo, impedance and air plethysmography) to reduce the complexity of the measurement and improve accuracy [30]. Using plethysmographic devices, information such as the venous filling index (VFI), ejection fraction, residual volume fraction (RVF) and arterial pulse wave shape can be determined and used to evaluate peripheral vascular function [35, 36]. Strain gauge, photo, impedance, and air plethysmography are discussed further in the following sections. Figure 1 represents an example of different plethysmography techniques.

### Strain-gauge plethysmography (SGP)

Strain gauge plethysmography (SGP) was first introduced in 1953 and later improved in the 1990s [30, 40–42]. SGP can be used to assess both the peripheral arterial and venous systems. The flexible strain gauge is filled with a conductive medium, usually mercury or gallium, and fit snugly around the limb (Fig. 1a) [31]. Changes in limb blood volume is estimated from proportional changes in the electrical impedance of the strain gauge [31].

Strain gauge plethysmography’s reliability has been questioned because of the lack of validation studies [31]. Reference values for computerized SGP were provided in a 2014 study based on data from 63 healthy controls and 56 patients with DVT and post-thrombotic changes [42]. Table 1 represents the mean and 95% confidence interval reference values for each variable (venous emptying, venous outflow rate, half refilling time and venous refilling volume) derived from the control and patient with DVT cohorts [42]. Venous emptying was defined as the blood volume reduction during the first second following cuff release. Venous outflow rate was defined as the expelled volume during the first 4 s following cuff release divided by the maximum venous volume and half refilling time referred to the time required for a 50% post exercise volume refilling [41, 42]. A right/left side difference of 5–10% was found to be normal for venous emptying and venous outflow rate. A 20–25% side difference for venous refilling volume and venous refilling time was also determined to be normal [42]. However, a reduction in venous emptying and venous outflow rate beyond these inferred a functional outflow obstruction, i.e. the presence of DVT [42].

**Table 1:** Computerized strain-gauge plethysmography reference values for controls and patients with DVT. from [42]

Venous parameter	Control (mean and 95% confidence interval)	Patients with DVT (mean and 95% confidence interval)
Venous emptying (mL/100 mL × min)	84 78–90	110 104–116
Venous outflow rate	0.58 0.54–0.62	0.76 0.74–0.78
Half refilling time (S)	6 4–7	17 15–18
Venous refilling volume (mL/100 mL)	1.11 0.95–1.27	1.65 1.52–1.78

Parameters indicative of DVT detection (venous emptying and venous outflow rate), muscle pump function (venous refilling volume), and the presence/absence of venous reflux (half refilling time) were tested for disease sensitivity [42]. Combined, these measures had an overall sensitivity of 96% to broadly detect a venous disorder, without the sensitivity to specifically identify the particular component (i.e. the presence of venous reflux and/or DVT) [42]. From these results, it is not possible to determine the accuracy of diagnosing specific venous disorders via strain gauge plethysmography. Other limitations that need to be considered with strain gauge plethysmography include temperature sensitivity and chemical hazard [43]. Adoption of indium gallium strain gauges instead of mercury can reduce this hazard [42].

### Photo plethysmography (PPG)

Photo plethysmography (PPG), first introduced in the 1930′s to assess the vascular system, uses an infrared light source and a light receptor to estimate the variation of blood volume [44]. There are two common PPG sensor designs; one in which the toe or finger is placed between a light source and a light receptor, commonly known as PPG (Fig. 1b); in the second configuration, known as quantitative PPG or light reflection rheography (LRR) (Fig. 1c), the light source and receptor are placed beside each other [31]. PPG produces a pulsatile waveform (AC) superimposed on a slowly changing baseline (DC) [45]. The AC component is used to measure changes in the blood volume due to arterial pulsation, and the DC component changes in total blood volume [31, 45, 46]. The derived arterial pulse wave enables the diagnosis of arterial incompetence [31]. Venous refilling time can also be measured by calculating changes in blood volume between static positioning and post-exercise (usually ten dorsiflexion manoeuvres) [31]. Venous refilling time is the time taken for the PPG curve to return to a stable value for at least 5 s [47]. Table 2 provides a summary of the sensitivity and specificity values calculated from evaluations of PPG for the diagnosis of PVDs.

**Table 2:** Sensitivity and Specificity evaluation for the use of photo-plethysmography (PPG) in the diagnosis of PVDs

Study	Diagnosis target	Reference method	N (control/patient)	PPG feature	Sensitivity	Specificity
Allen et al. [48]	PAD	ABI	107 (63/44)	PPG waveform	90.6%	88.9%
Ro et al. [49]	PAD	Angiography	194^a^ (31/163)	PPG waveform	81.6%	77.4%
Bays et al. [50]	CVI	Duplex ultrasound	20 (10/10)	PPG refill time (venous refill time)	100%	60%
Sarin et al. [51]	CVI	Duplex scanning	304^a^ (80/224)	PPG refill time (venous refill time)	74–79%	61%
Mitrani et al. [52]	DVT	Venography	69 (45/24)	Venous emptying (threshold 3 mm)	96%	71%
				Venous emptying (threshold 6 mm)	86%	89%
				Venous emptying rate (threshold 0.17 mm/s)	83%	89%
				Venous emptying rate (threshold 0.31 mm/s)	96%	78%
Arora et al. [53]	DVT	Venography	69^a^ (41/28)	Venous Emptying Rate (threshold 0.35 mm/s)	96.4%	82.9%
Thomas et al. [54]	DVT	Venography	131^a^ (61/70)	Shape of PPG trace	92%	84%
Tan et al. [55]	DVT	Venography or duplex	103^a^ (66/37)	Venous refilling time (threshold 20 s)	100%	47%
				Venous refilling time (threshold 36 s)	100%	35%
				Venous refilling time and venous pump	100%	56%

“Control” refers to the number of the subjects diagnosed without PVD by the reference method

“Patient” refers to the number of the subjects diagnosed with PVD by the reference method

^a^Denotes the number of the limbs used in the study and not the number of subjects

A study of 63 healthy subjects and 44 PAD patients compared PPG derived pulse wave analysis techniques extracted timing, amplitude and shape characteristics for both toes and for right-to-left toe differences and compared these to diagnosis using the Ankle Brachial Index (Table 2). Sensitivity of 90.6%, specificity of 88.9% and accuracy of 90.2% was reported [48]. In a later study, comparing PPG to angiography, a reduced sensitivity (81.6%) and specificity (77.4%) was reported in a cohort of 97 patients (194 legs; Table 2) [49]. In this study, a physician visually interpreted the average of PPG waveform during at least 60 heartbeats to diagnose PAD. Qualitative evaluation of the PPG waveform and greater precision of angiography over ABI may account for the lower reported specificity and sensitivity values [2, 56, 57].

PPG has also been used to evaluate venous reflux, with a reported sensitivity of 100% and specificity of 60%, in a relatively small study of 10 healthy subjects and 10 subjects with deep venous reflux diagnosed using duplex ultrasonography [50]. The venous refill time was calculated following five dorsiflexion manoeuvres with the leg in a dependent position while sitting [50]. The mean venous refill time for the healthy group was 20.2 ± 1.1 and 6.4 ± 8.9 s in the patient group [50]. A threshold venous refill time greater than 20 s was considered normal, In a larger study of 152 patients (304 legs) using the 20 s venous refill threshold; lower sensitivity 74% (superficial reflux), 79% (deep venous reflux) and specificity (61%) was reported [51].

Examinations of the clinical utility of PPG in the diagnosis of deep vein thrombosis (DVT) have reported sensitivity between 83 and 100% and specificity between 35 and 89% (Table 2) [48–51]. In a study of 69 patients with suspected DVT, PPG using LRR was compared with venography for differences in venous emptying rates following 10 ankle dorsiflexion exercises [52]. A venous emptying threshold (∆R) of ≤ 3 mm (measured from the LRR tracing) resulted in sensitivity of 96% and specificity of 71% for detecting a DVT. Increasing the ∆R threshold to ≤ 6 mm reduced sensitivity to 86% and increased specificity to 89% [52]. Alternatively, using the venous emptying rate to diagnose DVT, a threshold of 0.17 mm/s produced sensitivity of 83% and specificity of 89% [52]. Increasing the threshold to 0.31 mm/s, a sensitivity of 96% and a specificity of 78% was achieved [52]. Similarly in a study of 69 limbs a threshold value of 0.35 mm/s for the venous emptying rate, provided a sensitivity of 96.4% and a specificity of 82.9% [53]. Furthermore, the performance of LRR, in comparison to either venography or duplex imaging, in a study of 103 legs with a suspected DVT provided high sensitivity but without high specificity [55]. This study evaluated venous refilling time and venous pump function, defined as the amplitude of the LRR trace during dorsiflexion contraction [55]. A venous refilling time threshold of ≤ 20 s returned sensitivity of 100% and specificity of 47%. Increasing this threshold to ≤ 36 s, sensitivity remained the same but specificity reduced to 35%. The combined specificity of venous refilling time and the venous pump was still quite low at 56% [55]. These measures of venous refilling time and venous pump function progressively decrease with age, further leading to less distinction between normal and abnormal groups [55].

Thomas et al. used a different criterion for assessing the performance of LRR versus venography in a group of 131 legs with clinically suspected DVT, classifying a DVT by a flat or a virtually flat LRR trace [54]. Using the shape of the LRR trace as the diagnostic criteria, they achieved a sensitivity of 92% and a specificity of 84% [54]. They reported that false negatives or positives only occurred for patients aged ≥ 55 years, suggesting that LRR may not be a good screening tool in the elderly [54].

### Impedance plethysmography (IPG)

In 1939, Nyboer introduced the concept of impedance plethysmography (IPG), and in the 1970′s several IPG devices became commercially available particularly for the diagnosis of PVDs [36, 58]. IPG uses electrical impedance to derive changes in blood volume to determine hemodynamic functionality. In this method, circumferential electrodes are placed on the leg (Fig. 1d), a weak high-frequency alternating current passes through the leg, and voltage changes in the electrodes are monitored to measure blood volume changes in the test area [31]. This plethysmography method is reported to be less cumbersome than fluid displacement based plethysmographic methods [53, 54]. Additionally, as discussed in the following paragraphs, IPG can be employed to detect DVT and to evaluate both arterial and venous competence [36, 59, 60].

In a study of 33 legs with arteriography confirmed arterial obstruction greater than 50% diameter, compared to 28 healthy control legs, IPG waveform analysis (resting arterial pulse wave amplitude and maximum systolic slope) provided a sensitivity and specificity greater than 90% [36]. Threshold pulse wave amplitude of 0.06% dR (resistance change due to blood volume change) and 0.60% dR/s for systolic slope were used [36]. When compared to ABI diagnosed PVD in a study of 66 patients, a sensitivity of 73.2% and a specificity of 96% was reported, using crest time thresholds of 180 ms [61].

Anderson evaluated IPG for detecting CVI in 44 subjects compared to Doppler ultrasound [36]. Venous refilling time > 11 s was chosen to represent a competent set of venous valves, while < 11 s indicated venous reflux [36]. Reported accuracy was 90%, however, sensitivity and specificity values were not provided [36].

**Fig. 2:**
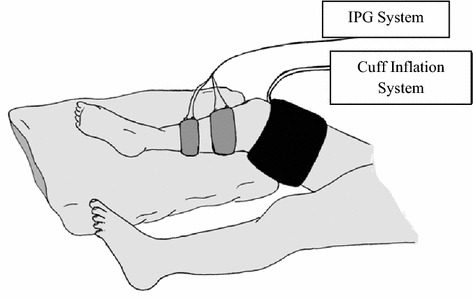
Schematic view of the use of impedance plethysmography for detection of DVT (adapted from [36])

Impedance plethysmography to detect the presence of DVT evaluates the patient in supine position with the leg elevated above the level of the heart (Fig. 2). A thigh cuff is inflated above venous pressure (50 mmHg) with calf impedance monitored until plateau before cuff deflation. The presence of DVT is inferred by measuring maximum venous capacitance (venous volume after 50 mmHg pressure application) and venous outflow rate (venous volume decrease in the first 3 s of deflation) [36, 62]. Six medical centres were cited to independently confirm the accuracy (> 94%) of IPG to diagnose a recent DVT proximal to the knee [36]. Sinton et al. compared IPG with venography in 85 legs and reported that IPG was successful in detecting proximal DVT in 20 of 22 subjects [63]. However, IPG was less successful in distinguishing been healthy and pathological groups for distal DVT’s [63]. A review of venous disorder diagnosis reported acceptable sensitivity (87–98%) for IPG compared to venography [62, 64–69]. In contrast, sensitivity (12–64%) was less acceptable in asymptomatic populations [62, 70–73].

### Air plethysmography (APG)

Air plethysmography (APG) was first introduced by Christopoulos et al. in 1987 for evaluation of venous function in the lower extremities [74, 75]. An air-filled chamber encloses the lower limb and air displacement is used to measure blood volume changes (Fig. 1e). The venous filling index (VFI) measurement involves the patient elevating their leg in the supine position, allowing the leg to empty, and then resuming the standing position. The VFI is defined as 90% of the venous volume at rest divided by the time taken for 90% venous refilling following the leg elevation manoeuvrer [75]. VFI has been reported to be predictive of venous reflux, with a VFI less than 2 mL/s demonstrated no reflux, legs with VFI of 2–7 mL/s demonstrated reflux to above the knee, and a VFI higher than 7 represented venous reflux to below the knee [74]. A VFI threshold greater than 7 had sensitivity of 73%, specificity of 100%, and accuracy of 81% when compared to phlebography for diagnosing venous reflux in 31 limbs [74].

In a study of 186 extremities, APG derived VFI demonstrated sensitivity of 80% and specificity of ~ 94% for detecting venous reflux when compared to venography and duplex scanning results [35]. The investigators also calculated residual volume fractions − difference between venous volume in the leg elevation position and following ten heel raise exercises, and divided by venous volume at rest [35, 75, 76]. Another study also showed that using residual volume fraction can increase the specificity from 90 to 100% compared to VFI [50]. While residual volume fractions can increase specificity, it is not very helpful clinically as only a small subset of patients with symptomatic venous insufficiency have a normal VFI [35]. Due to the relative ease-of-use and reproducibility of APG, it has replaced SGP and PPG in CVI diagnosis [74].

Air plethysmography can also be used to detect the presence of venous occlusion. Venous obstruction affects the relationship between venous volume and pressure [74, 77]. Harada et al. used APG to measure residual volume fraction and compared it with ambulatory venous pressure [74]. A high correlation (r = 0.86) between these two factors was found when no venous obstruction was present, while a poor correlation (r = 0.40) indicated venous occlusion [74]. However, in terms of clinical utility, the measurement of ambulatory pressure is an invasive and uncomfortable procedure, making this diagnostic unsuitable for routine use.

#### Summary and limitations

Several plethysmography methods have been developed since 1677 for the assessment of peripheral vascular function noninvasively. Table 3 provides a summary of the discussed plethysmography techniques. The oldest plethysmography method, water plethysmography, has been used mostly in clinical research rather than in medical practice, as it is cumbersome to use and difficult to calibrate [31]. Later, strain gauge and photo plethysmography were introduced. Strain gauge plethysmography lacks validation studies [31], is sensitive to temperature variations, and the use of mercury gauges have environmental pollution concerns [43]. On the other hand, while photo plethysmography has acceptable sensitivity and specificity for arterial disease, it has low sensitivity and specificity when detecting venous reflux [51], which further decreases in elderly populations [54]. Impedance plethysmography and air plethysmography were developed later. However, impedance plethysmography has shown to have low sensitivity (12 to 64%) for detecting asymptomatic DVTs [62] and air plethysmography has a relatively low sensitivity (73%) in detecting venous reflux [74]. Additionally, air plethysmography requires associated use of invasive ambulatory venous pressure measurement for diagnosis of venous occlusion. Therefore, although air plethysmography has been reported to have more reproducibility than strain-gauge plethysmography and photo-plethysmography [74], it is not the most popular technique for peripheral vascular assessment. Photo-plethysmography is generally most popular, however, plethysmography remains cumbersome, requires careful setup by a trained practitioner, and takes considerable time to perform the assessment [14]. While plethysmographic methods have considerable utility in functional assessment, these time and personnel requirements have meant that they have been largely replaced by Doppler ultrasound methods which will be discussed in the next section [31].

**Table 3:** Selection of studies using plethysmography methods in the diagnosis of PVDs

Study	Diagnosis target	Reference method	Number of subjects (control/patient)	Plethysmography method	Sensitivity	Specificity
Harada et al. [74]	CVI	Venography	31^a^ (0/31)	APG	73%	100%
Criado et al. [35]	CVI	Duplex scanning and venography	186^a^ (61/125)	APG	80%	94%
Bays et al. [50]	CVI	Duplex ultrasound	20^a^ (10/10)	APG	70%–100%	90%–100%
Rosfors et al. [42]	Venous disorder (CVI, DVT)	Duplex ultrasound	119 (63/56)	SGP	96%–98%	Not given
Allen et al. [48]	PAD	ABI	107 (63/44)	PPG	90.6%,	88.9%
Ro et al. [49]	PAD	Angiography	194^a^ (31/163)	PPG	81.6%	77.4%
Bays et al. [50]	CVI	Duplex ultrasound	20^a^ (10/10)	PPG	100%	60%
Sarin et al. [51]	CVI	Duplex scanning	304^a^ (80/224)	PPG	74–79%	61%
Mitrani et al. [52]	DVT	Venography	69 (45/24)	PPG	83%–96%	71%–89%
Arora et al. [53]	DVT	Venography	69^a^ (41/28)	PPG	96.4%	82.9%
Thomas et al. [54]	DVT	Venography	131^a^ (61/70)	PPG	92%	84%
Tan et al. [55]	DVT	Venography or duplex	103^a^ (66/37)	PPG	100%	35%–56%
Anderson [36]	PAD	Angiography	61^a^ (28/33)	IPG	> 90%	>90%
Mašanauskienė et al. [61]	PAD	ABI	62 (21/41)	IPG	73.2%	96%
Hull et al. [64]	DVT	Venography	200 (114/86)	IPG	94%	91%
Toy et al. [65]	DVT	Venography	25^a^ (9/16)	IPG	94%	100%
Flanigan et al. [66]	DVT	Venography	207^a^ (136/71)	IPG	90.4%	75.7%
Cooperman et al. [67]	DVT	Venography	98^a^ (75/23)	IPG	87%	96%
Liapis et al. [68]	DVT	Venography	308^a^ (169/139)	IPG	91%	89%
Peters et al. [69]	DVT	Venography	185 (124/61)	IPG	84%	93%
Cruickshank et al. [70]	DVT	Venography	1010^a^ (812/198)	IPG	12.9%	98.1%
Paiement et al. [72]	DVT	Venography	937^a^ (864/73)	IPG	12.3%	99.1%
Agnelli et al. [73]	DVT	Venography	440^a^ (295/145)	IPG	19%	91%

“Control” refers to the number of the subjects diagnosed without PVD by the reference method

“Patient” refers to the number of the subjects diagnosed with PVD by the reference method

^a^Denotes the number of the limbs used in the study and not the number of subjects

## Doppler ultrasound methods

Vascular ultrasonography is a non-invasive diagnostic method utilizing a handheld transducer to direct an inaudible sound wave with a frequency of 1–30 MHz into the vessel of interest to assess vascular competency [78]. Computer processing converts the data to audible sound waves or graphs, allowing the vascular technician to see and/or hear blood flow through the vessels and is the mainstay of vascular imaging. B-mode, continuous wave, pulsed wave Doppler and duplex ultrasound are used to assess the vascular system [28, 78–81].

### B-mode Doppler ultrasound

B-mode (brightness mode) ultrasonography or grey-scale imaging generates a two dimensional real-time picture to visualize the structure of blood vessels and provides information about diameter change in large veins [50, 79, 82, 83].

Dauzat et al. evaluated the clinical value of B-mode for DVT detection in a group of 145 patients and reported a sensitivity of 94% and a specificity of 100% compared to venography [84]. O’Leary et al. also used B-mode and found a sensitivity of 88% and a specificity of 96% to detect DVT in 50 subjects compared to venography results [85]. In another study, Sullivan et al. compared B-mode ultrasound with venography for detection of DVT in 23 patients and found a sensitivity of 100% and a specificity of 92% [86]. They also compared the results of B-mode and rheography for detection of DVT in 170 extremities and found an agreement of 82% between the two methods [86]. This study also reported the capability of B-mode in distinguishing an acute DVT from a chronic DVT in 93% of the extremities which underwent venography [86].

### Continuous wave (CW) Doppler ultrasound

Continuous wave (CW) Doppler ultrasound is routinely used for clinical screening. Venous flow is heard as a low-pitched blowing sound and a normal venous blood flow should be in phase with respiration [78, 80]. If manual limb compression is applied distal to the probe, the forward flow (flow towards the heart) will be augmented. The augmentation can be seen as an increase in the amplitude of the CW Doppler signal [78]. If limb compression is applied proximal to the probe, and valves are competent, the Doppler signal should cease as healthy valves limit retrograde flow [78]. A similar decrease in the blood flow signal can be noted when the patient coughs or performs a valsalva manoeuvre [78]. Venous reflux can thus de diagnosed by an audible signal during the compression or valsalva manoeuvre. If the signal lasts for more than 5 s, venous reflux is inferred [78, 82].

### Pulsed wave (PW) Doppler ultrasound

Pulsed wave Doppler mode can be used for categorizing peripheral arterial stenosis [81]. Although PW Doppler overcomes the limitation of CW Doppler in depth discrimination, it is unable to measure high velocities due to the aliasing phenomenon [87]. Aliasing occurs when the velocity of blood flow exceeds one half of the pulse repetition frequency (usually a velocity above 2 m/s) and it affects the velocity waveform such that the velocity and direction of blood flow cannot be interpreted [87].

### Duplex ultrasound (DU)

Duplex ultrasound combines the use of B-mode imaging, PW, CW Doppler modes when evaluation of the anatomy and hemodynamic function of the vascular system is needed [28]. Duplex ultrasound thus is a very sensitive diagnostic method [82], and can be used for detecting venous reflux, arterial stenosis/occlusion and deep vein thrombosis. In one study, 169 limbs were evaluated using duplex ultrasound for diagnosing peripheral arterial disease [88]. Patients rested for 15 min before examination. Subsequently, both lower limbs were scanned from the common femoral artery to the pedal arteries and the entire limb was divided into 15 segments [88]. Arterial occlusion was determined by observation of a dampened distal signal in comparison with the proximal signal; presence of a proximal exit collateral; and presence of a distal re-entry collateral. Peak systolic velocity ratio (the peak systolic velocity in the stenosis divided by the peak systolic velocity just proximal to the stenosis) was also measured for those segments with flow velocity increase [88]. Segments with a peak systolic velocity ratio ≥ 2 m/s inferred a diameter reduction ≥ 50% [88]. Duplex ultrasound was reported to have a sensitivity of 88%, specificity of 79% and accuracy of 95% among 2535 segments (169 limbs × 15 segments) when compared to angiography (Table 4) [88]. In another study of 100 subjects, greater sensitivity 95 and 92% and specificity (99 and 97%) were reported for the diagnosis of arterial occlusion and stenosis respectively when compared to angiography [28, 89]. A review of Duplex ultrasound performance for the diagnosis of PAD found the sensitivity values between the range of 79.7–97% and the specificity ranged from 88.5 to 99% in comparison to angiography [90–93]. It also suggested that segment-to-segment comparison possibly increases the number of true negative test results leading to an overestimation of the specificity [90].

**Table 4:** Selected studies using Doppler ultrasound methods in diagnosis of PVDs

Study	Diagnosis target	Reference method	Number of subjects (control/patient)	Doppler ultrasound method	Sensitivity (%)	Specificity (%)
Dauzat et al. [84]	DVT	Venography	145 (45/100)	B-mode	94	100
Sullivan et al. [86]	DVT	Venography	23^a^ (12/11)	B-mode	100	92
O’Leary et al. [85]	DVT	Venography	50 (25/25)	B-mode	88	96
Cronan et al. [96]	DVT	Venography	51 (23/28)	Duplex	89	100
Aly et al. [92]	PAD	Angiography	177^a,b^	Duplex	92	99
Linke et al. [91]	PAD	Angiography	46^a,b^	Duplex	89	95
Bergamini et al. [93]	PAD	Angiography	80^a^ (28/52)	Duplex	80	95
Eiberg et al. [88]	PAD	Angiography	169 (0/169)	Duplex	88	79
Whelan et al. [89]	Arterial occlusion	Angiography	51 (8/43)	Duplex	95	99
Whelan et al. [89]	Arterial stenosis	Angiography	51 (8/43)	Duplex	92	97

“Control” refers to the number of the subjects diagnosed without PVD by the reference method

“Patient” refers to the number of the subjects diagnosed with PVD by the reference method

^a^Denotes the number of the limbs used in the study and not the number of subjects

^b^Comparison between limb segments and not control/patients

Duplex ultrasound is arguably the most important and widely used non-invasive tool for the investigation of chronic venous diseases [94]. It can detect minimal venous reflux even in isolated veins of asymptomatic individuals [82]. Duplex scanning can also determine if the reflux is constrained in veins above or below the knee [82], a limitation of the previously reported IPG diagnosis of venous reflux. Duplex ultrasound scanning is undertaken with the patient in the standing position to allow maximum venous dilation [95]. Several manoeuvres such as foot/calf compression, ankle dorsiflexion and Valsalva can be performed to create physiologic flow [94]. Alternately the patient may be placed in a 15° reversed trendelenburg position and then asked to perform a Valsalva manoeuvre [82]. Nicolaides argued that both of these examination lead to similar results, and while the second method is more convenient, it does require cooperation from the patient [82]. A reversal of flow during the diagnostic manoeuvres infers venous reflux [94]. Threshold values for the diagnosis of venous reflux are defined as retrograde flow lasting longer than 1000 ms in the femoral area or longer than 500 ms in the femoral and popliteal veins [95].

At the beginning of the 21st century, the use of duplex ultrasound was extended to detect venous obstruction and its extent [82]. Continuous flow in the femoral veins with little or no change in flow during any manoeuvre infers abnormality [95]. However, the presence of phasic flow does not exclude the potential presence of an obstruction and repeat scans are recommended to confirm or exclude DVT [95]. While duplex ultrasound can detect the presence of venous stenosis by measuring luminal reduction the extent of occlusion is better evaluated with magnetic resonance venography, computed tomography venography or contrast venography [95]. Cronan et al. compared the performance of Duplex ultrasound for detection of DVT in 51 subjects and found 89% sensitivity and 100% specificity [96]. In a systematic review of diagnostic accuracy of ultrasound for DVT, sensitivity of Duplex ultrasound ranged from 75 to 96%, and a specificity of 94% depending on the site of DVT [97].

Colour duplex imaging can also be used to evaluate the direction and velocity of blood flow and thus detect the location of arterial occlusion/stenosis and venous reflux [28, 81, 98, 99]. A study compared colour Doppler imaging with angiography for detection of occlusion and stenosis in one hundred legs of 51 patients [89]. Occlusion detection had a sensitivity of 95% and specificity of 99%, while stenosis detection had sensitivity of 92% and a specificity of 97% [89]. Examination time of 30–45 min was reported for each patient [89]. In general, colour duplex imaging provides better accuracy [98]. However, Doppler transducer positioning (70° to the vessel) is critical and requires highly trained and experienced operators [100].

#### Summary and limitations

Vascular ultrasonography is one of the most commonly used non-invasive methods employed by vascular laboratories to define anatomy, hemodynamic and lesion morphology. Ultrasound examination is considered to be the gold standard and a very powerful tool in establishing diagnosis and aiding therapeutic management of chronic venous insufficiency and peripheral arterial disease, revealing sites of reflux and/or obstruction in the venous system, arterial occlusions and stenosis [95]. However, the use of duplex ultrasound is highly operator dependent [82, 88]. Furthermore, 5–20% of patients cannot undergo duplex ultrasound wave exposure because of ulceration, pain, swelling, heavily calcified arteries and obesity [88]. Moreover, duplex ultrasonography can be time-consuming (1–2 h for full assessment), requires expensive equipment and a highly trained, experienced vascular technician with comprehensive knowledge of the anatomy of the vascular system [94]. Lack of a universally accepted protocols for detection of DVT using the ultrasonic methods is an additional issue [101]. These factors therefore limit the use of ultrasound for routine examination and early diagnosis of PVDs.

## Blood pressure measurement methods

### Ankle Brachial Index (ABI)

In the 1950′s, Winsor first described the Ankle Brachial Index (ABI), a simple non-invasive method for assessing arterial perfusion [102]. It remains a primary clinical diagnostic test for PAD [103]. The ABI is measured by calculating the blood pressure at the ankle and dividing by the higher of two brachial systolic blood pressures [28, 102]. A normal ABI is between 1 and 1.3 [104] with 0.91–0.99 acceptable [103]. An ABI lower than 0.9 indicates the presence of PAD with ratios below 0.4 indicating the presence of severe PAD and problems for healing [28, 105]. While an ABI between 0.91 and 0.99 is acceptable, this range and below also indicates increased cardiovascular risk [105], including stroke, coronary diseases or cardiovascular death [106–108]. ABI has a relatively high sensitivity and specificity, but such high accuracy cannot be achieved for all patient types. Arteries of the elderly, patients with diabetes or renal disease are usually calcified and largely incompressible, leading to poor sensitivity in such cases [28]. The poor sensitivity of ABI has been referenced in studies where the ABI appeared to be normal (1–1.3) or even supernormal (above 1.3) for a group of patients with PAD [28, 109].

A single ABI measurement may not be sufficient for diagnosis even in symptomatic cases [105]. In such cases, the patient is asked to perform a standardized exercise, after which a ABI is recalculated [105]. Many vascular laboratories use a standardized exercise protocol [110], this may involve treadmill walking at a 12-degree incline, at 2 mph, for at least 5 min or graded bike pedalling [105]. Decreases in post exercise ankle pressure of 20 mmHg or more is indicative of severe PAD [105]. While the ABI is a simple test, it can be time consuming and requires training and experience [15]. A recent review highlighted the importance of training by comparing sensitivity and specificity of oscillometric ABI and manual Doppler ABI performed by inexperienced operators [104]. While oscillometric ABI provides sensitivity of 97% and specificity of 89%, manual Doppler ABI has sensitivity of 95% and specificity of just 56% compared to angiography [104, 111]. Xu et al. reviewed sensitivity and specificity of ABI in detecting/excluding PAD and found the sensitivity values between 61–96% and the specificity range within 56–90% [56, 57, 112–118]. An ABI test typically takes about 15 min [119], and should be preceded by a 30-min rest period [56]. While ABI is useful as an initial clinical test to assist diagnosis, not all guidelines promote the ABI as a screening tool for PAD in primary care [16]. The ABI is unable to identify the location of arterial stenosis/occlusion [28], is not recommended as a PAD screening tool in primary care by all guidelines [16] and is not capable of diagnosing CVI or DVT.

### Segmental blood pressure measurement

Segmental blood pressure measurement, unlike ABI measures, can be used to localize the site of stenosis or occlusion in PAD [28, 105, 120, 121]. Four cuffs are placed around the leg, ankle, calf lower thigh and upper thigh [120, 121] with either handheld Doppler ultrasound, photoplethysmography, strain-gauge plethysmography, or oscillometric blood pressure measurement then used to measure the blood flow/pressure at each of the four leg cuff sites [105, 120].

A reference arm blood pressure measure is also taken and normally is at least 30 mmHg lower than thigh pressure [105, 120, 122]. In healthy individuals the pressure difference (gradient) between two adjacent levels in the lower extremities should be 20 mmHg or less [81, 105]. A pressure gradient of 20–30 mmHg is representative of stenosis with greater pressure reductions indicating occlusion [120, 123, 124]. A pressure difference ≥ 20 mmHg between the bilateral leg segments also indicates arterial occlusion [121].

Limitations of this method include inappropriate cuff sizing resulting in false blood pressure readings [120]. An average error of 8.5 mmHg in systolic blood pressure is reported due to inappropriate cuff sizing [120, 125]. Accuracy can be improved by including other measures such as arterial pulse wave analysis [105], although no documented accuracy values are available.

### Toe Brachial Index (TBI) method

Toe blood pressure measurements to evaluate peripheral arterial disease was introduced in 1965 [124, 126] and is particularly common for the diagnosis and management of underlying vascular pathology associated with diabetic foot lesions [126–128]. Similar to ABI, TBI is calculated by dividing the toe systolic pressure by the brachial pressure. TBI is recommended as an alternative to ABI to counter unreliable elevated measures in the presence of medial artery calcification, which is particularly prevalent in people with diabetes and the aged [129–131].

A study of 174 subjects with diabetes and 53 non-diabetic subject found that diabetic patients with an ABI < 1.3 had ABI–TBI differences within the normal ranges for healthy controls, whereas those with an ABI ≥ 1.3 had abnormal ABI–TBI differences [129]. The authors suggest that there is no advantage to TBI over ABI where ABI < 1.3 but that the TBI is superior in the presence of calcification, where ABI ≥ 1.3 [28, 129]. While exact threshold values for TBI are still debated, a TBI ≥ 0.7 is generally reported to be normal with a TB < 0.7 associated with claudication and a TBI < 0.2 with pain at rest [28].

A review of 22 studies [130] found TBI threshold levels to indicate PAD ranging from 0.54 to 0.75 [118, 129, 132–152]. Despite this range in TBI threshold values, TBI has high sensitivity (90–100%) compared to angiography, with specificity values between 65 and 100% [130]. Several guidelines have suggested using TBI < 0.7 as the threshold, however, based on Høyer et al.’s review this cutoff is not necessarily evidence-based. According to Høyer et al.’s findings, there is a lack of agreement on TBI diagnostic threshold in the current literature and more trials are required to recommend the best diagnostic threshold [130]. Test environments and protocols are important to improve test performance. The patient is required to rest for at least 5 min before the measurement, the room temperature should be above 22 °C and toe skin temperature ≥ 25 °C [126]. Pre-test limb heating may be required, in order to minimize false positive results [130, 153, 154].

The measurement of toe blood pressure is technically more complicated than measuring ankle blood pressure [129]. The additional equipment required, such as photo-plethysmography, strain gauge plethysmography and Doppler flowmeter, can limit use in some clinical settings [129, 130].

#### Summary and limitations

Although the ABI is relatively cheap, requiring minimal and inexpensive equipment, and is widely clinically applied, it has a low sensitivity when used in patients with diabetes or the elderly with calcified arteries, where the ABI values are inflated mimicking false negative normal values [28]. This is especially troublesome as these two groups of patients are at higher risk of developing PVDs. TBI can be used as an alternative in cases with the presence of medial artery calcification, but adds an additional time constraint. To assist in pathology site location additional segmental blood pressure measurements can be used. Table 5 presents a summary of the discussed blood pressure measurement methods.

**Table 5:** Selected studies using blood pressure measurement methods in diagnosis of PVDs

Study	Diagnosis target	Reference method	Number of subjects (control/patient)	Blood pressure measurement method	Sensitivity	Specificity
Vega et al. [111]	PAD	Angiography	158^a^ (27/131)	ABI	95%–97%	56%–89%
Wikström et al. [56]	PAD	Angiography	533^a^ (421/112)	ABI	15%–20%	99%
Parameswaran et al. [115]	PAD	Doppler waveform analysis	114^a^ (79/35) type 2 diabetes	ABI	63%	97%
Lijmer et al. [117]	PAD	Angiography	106^a^ (0/106)	ABI	79%	96%
Schröder et al. [112]	PAD	Duplex Ultrasound	216 (103/113)	ABI	68%	99%
Niazi et al. [113]	PAD	Angiography	208^a^ (42/166)	ABI	68%	83%
Guo et al. [114]	PAD	Angiography	298 (277/21)	ABI	91%	86%
Premalatha et al. [116]	PAD	Duplex ultrasound	94 (26/68) type 2 diabetes	ABI	70.6%	88.5%
Williams et al. [118]	PAD	Duplex ultrasound	41^a^ (27/14)	ABI	83%	100%
Williams et al. [118]	PAD	Duplex ultrasound	32^a^ (25/7) Diabetes	ABI	100%	88%
Williams et al. [118]	PAD	Duplex ultrasound	57^a^ (41/16) Diabetes with neuropathy	ABI	53%	95%
Williams et al. [118]	PAD	Duplex ultrasound	41^a^ (27/14)	TBI	100%	81%
Williams et al. [118]	PAD	Duplex ultrasound	32^a^ (25/7) Diabetes	TBI	91%	65%
Williams et al. [118]	PAD	Duplex ultrasound	57^a^ (41/16) Diabetes with neuropathy	TBI	100%	61%
Park et al. [152]	PAD	Angiography	30^a^ (17/13) (Diabetes gangrene)	TBI	100%	100%

“Control” refers to the number of the subjects diagnosed without PVD by the reference method

“Patient” refers to the number of the subjects diagnosed with PVD by the reference method

^a^Denotes the number of the limbs used in the study and not the number of subjects

## Emerging techniques

### Pulse wave velocity (PWV)

Pulse wave velocity is defined as the velocity at which the arterial pulse wave, generated by heart contraction, propagates through the arteries [155]. The application of pulse wave velocity as a measure of arterial stiffness/elasticity was first predicted by Thomas Young in 1808 [156]. Later, Moens and Korteweg independently presented a mathematical model indicating the relationship between pulse wave velocity and arterial stiffness [157]. Based on the Moens-Korteweg equation, $PWV = \sqrt {E.h/2\uprho.{\text{r}}}$ (E: intrinsic stiffness of the wall of artery, h: thickness, r: radius, ρ: blood density), higher PWV is representative of increased stiffness of arteries [157, 158]. Patients with PAD are reported to have a higher aortic PWV compared to healthy controls [159]. However, PAD is not the only potential cause of arterial stiffness, as arteries may become stiffened in the presence or absence of PAD, e.g. hypertension and diabetes [155].

Although the use of PWV as a diagnostic measure is quite old, its use for the diagnosis of PAD is relatively new. In two studies with sample sizes of 105 and 440 healthy subjects and 35 and 38 subjects with PAD, PWV was reduced in the presence of PAD when measured between the heart-feet [160] and femoral-dorsalis [161]. However, increased heart-feet PWV was observed in patients with hypertension, suggesting that PAD and hypertension apply opposing effects on PWV [160]. Even beat to beat blood pressure variability have shown to be correlated with an increase in PWV in the hypertensive population [162]. Additionally, in a study of 101 healthy subjects and 102 patients with diabetes with/without PAD, reduced brachial-ankle PWV (baPWV) was found in people with diabetes and PAD (1221 cm/s) compared to non-PAD diabetic subjects (1607 cm/s) [163]. The median difference between absolute right-left baPWV was 36 cm/s in the healthy group, 55 cm/s in the diabetic/no PAD group and 290 cm/s in the diabetic/PAD group suggesting the right-left difference may be used as a novel indicator of PAD [163]. However, cautious interpretation is needed when bilateral PAD is present and comparisons to ABI measures which have poor sensitivity in the presence of calcification [163].

The application of PWV as a measure of arterial stiffness has been motivated by the development of new devices to assist measurement. In a recent review PulsePen (DiaTecne, Milan, Italy), Complior (Colson, France), SphygmoCor (AtCor Medical, Sydney, Australia), photoplethysmography, ultrasound and magnetic resonance imaging (MRI) have been identified for non-invasive PWV measurement [164]. A number of other optical devices are identified but are not commercially available [164]. While a promising area of inquiry, PWV measurement is currently not used for clinical diagnosis of PVD. Limitations include the expense of ultrasound and MRI methods and poor accuracy in more affordable alternatives [164]. As discussed earlier, PAD is not the only parameter that alters PWV. The use of PWV as an indicator of PAD is complicated by the effects of ageing, arterial stiffness, hypertension, beat to beat blood pressure variability, and diabetes. Considering the work to date, it is still possible that PWV could be used for PAD diagnosis. However, no studies have compared PWV with validated diagnostic techniques; consequently, no values of sensitivity or specificity are reported.

### Vascular optical tomographic imaging (VOTI)

**Fig. 3:**
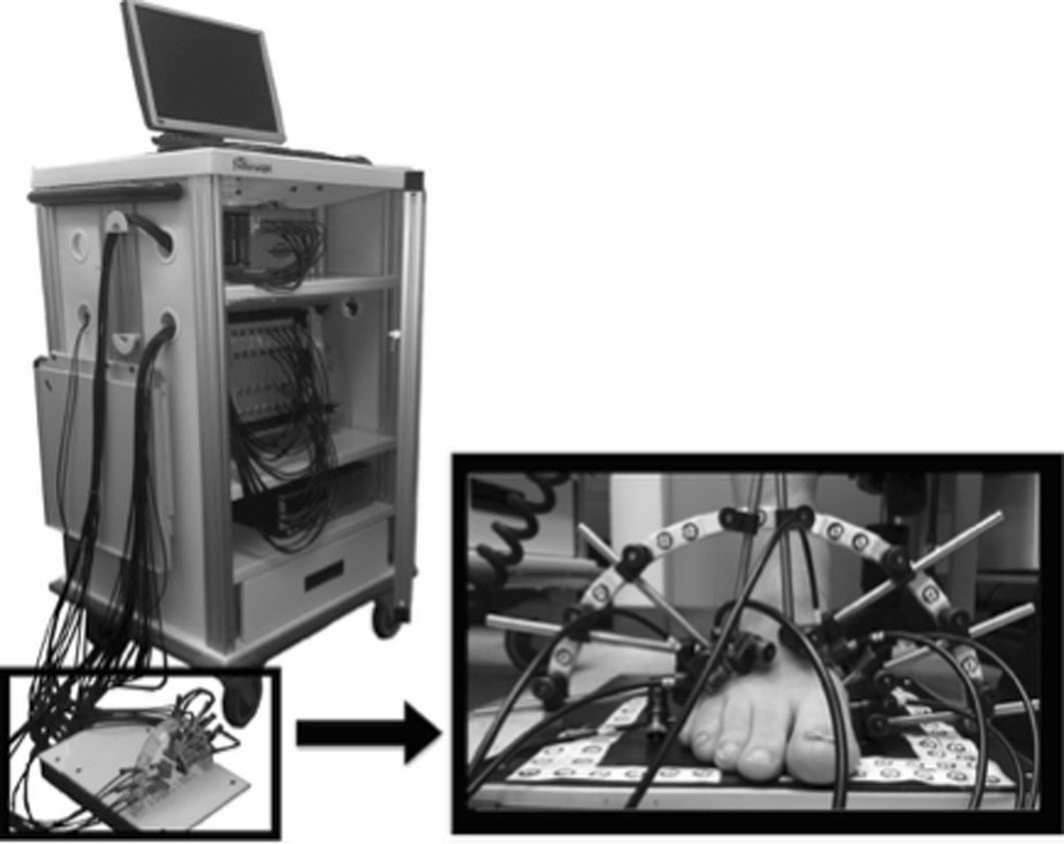
VOTI system and its sandal shaped measuring probe [165]

Vascular optical tomographic imaging (VOTI) is a new non-invasive imaging system, which can be used to directly measure distal perfusion in the foot by extracting information about haemoglobin concentration [165]. Although VOTI has not been routinely used in vascular clinics yet, this system has the potential to be used as a new diagnostic tool for peripheral arterial disease [165]. The VOTI system has a sandal shaped measuring probe encompassing the foot (Fig. 3), which uses harmless red, and near infrared light (650 nm < wavelength < 900 nm) to illuminate the foot at different points.

The system uses the transmitted lights to construct a cross-section image of haemoglobin concentration at the mid metatarsal region. Lower light intensities are indicative of higher haemoglobin concentration, which is itself representative of better perfusion [165].

The VOTI system was originally introduced in 2007 and in 2015 the system was used to assess PAD in the lower extremities of a group of 40 subjects [165]. Subjects were classified into healthy (n = 20), PAD (n = 10) and PAD with diabetes (n = 10) groups according to a combination of the patients’ ABI readings, segmental ultrasound results, physical symptoms, and medical history [165]. Haemoglobin concentration was extracted during staged occlusion and compared with ABI readings and arterial pulse wave recordings. Haemoglobin concentration for healthy subjects was found to be about twice that of patients during 60 mmHg venous occlusion, with a faster response to pressure application/release in healthy subjects compared to patients [165]. Despite dissimilar ABI readings from PAD patients and PAD patients with diabetes, similar haemoglobin time traces were observed in both cohorts, suggesting VOTI may have greater sensitivity in people with diabetes and PAD [165].

A peak haemoglobin concentration threshold of 6.1% during 60 mmHg venous occlusion resulted in sensitivity of 85%, specificity of 73% and accuracy of 80% in detecting PAD [165]. The required time for imaging the foot was reported to be about 15 min, however, time for the haemoglobin concentration image reconstruction, image processing, and peak value extraction is not given [165]. To date, no information about the training requirements or cost of the equipment have been released.

### Polymer-based sensors

Recently, the use of polymer-based sensors has shown potential for diagnosis of PVDs. Such sensors basically consist of two main components, a highly elastic polymer carrier (e.g. rubber) and an integrated conductive element (e.g. carbon). Typically, deformation of the sensor generates proportional changes in the impedance of the sensor.

**Fig. 4:**
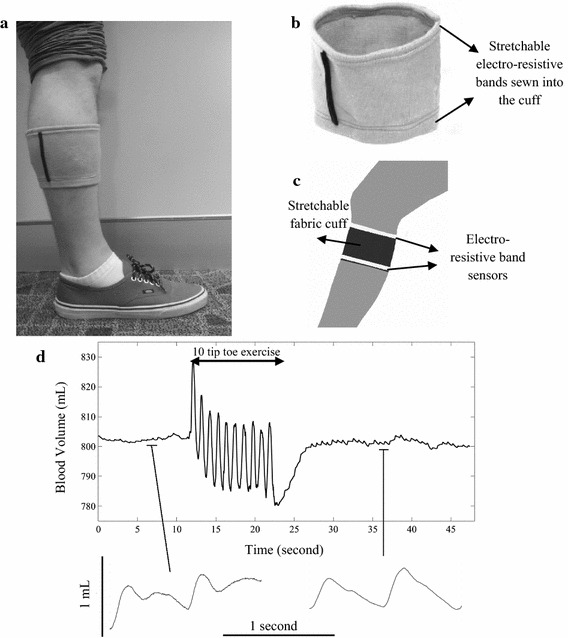
HeMo prototype; **a** a demonstration of HeMo worn on the calf [166]; **b** HeMo cuff (adopted from  [166]); **c** diagram of HeMo worn on the calf (adopted from [166]); **d** blood flow variations recorded before, during and after tiptoe exercise by HeMo [167]

In 2014, Breen et al. embedded commercially available Conductive Rubber Cord Stretch Sensors (available from Adafruit, NY, USA), also known as Electro Resistive Bands (ERB), in a piece of stretchable fabric (Fig. 4) to visualize peripheral blood flow [166]. The carbon-black impregnated rubber ERB sensors, have fixed resistance at rest, with impedance increasing when stretched. When worn around the calf (Fig. 4a) changes in leg volume in the leg can be monitored [166, 167]. This new device, HeMo (Hemodynamic Monitor), is sensitive to both postural changes and arterial inflow allowing capture of arterial pulse wave and venous filling index (Fig. 4d) [166, 167]. HeMo has been compared with a commercially available plethysmography device, VasoScreen 5000 (medis, Ilmenau, Germany), demonstrating the capability of the prototype to assess both peripheral arterial and venous competence in the leg [168]. HeMo is a easy to use, low cost device that potentially will require only limited training [166–168]. Moreover, HeMo recordings are calibrated in mL allowing the monitoring of progression or treatment of PAD over time [167]. However, this technology is at an early stage, trials prepared according the most updated ethical policies [169, 170] are scheduled for late 2018, hence no sensitivity or specificity values are reported to date and no sensitivity or specificity values are reported to date.

Boland et al. introduced two new polymer-based sensors, one created by infusing exfoliated graphene into natural rubber [171] and another, G-putty, made by adding graphene to commercially available silicon polymer (Silly Putty, Crayola, Easton, PA) [172]. Both of these sensors are highly stretchable and capable of capturing arterial pulse waves [171, 172]. These graphene polymer composites are low cost and can provide better sensitivity and extensibility (performance beyond 800% strain) than carbon black polymer sensors [171]. However, neither the graphene-rubber strain sensor nor G-putty is yet commercially available.

## Discussion and conclusion

This review discussed the current non-invasive hemodynamic monitoring techniques and demonstrated their potential for the non-invasive assessment of peripheral vascular function. A summary table including the reviewed methodologies together with their performance (where available) in terms of sensitivity, specificity and accuracy is associated with this paper as Additional file 1: Table S1.

**Fig. 5:**
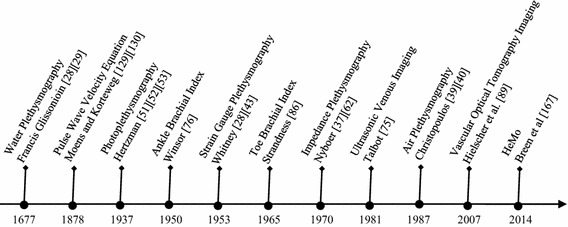
Advent of milestone technologies for noninvasive diagnosis of PVDs

Considering the prevalence and the adverse impacts of PVDs, it is no surprise that diagnostic methods have been developed since the 1670s. The timeline in Fig. 5 shows the advent of milestone technologies for non-invasive diagnosis of PVD. Despite the efforts in the development of an ideal non-invasive diagnostic modality for PVDs, underdiagnosis in the primary care is still an ongoing challenge, which demands development of new unobtrusive monitoring techniques [173–175].

Plethysmographic methods have the best utility to detect both arterial and venous disease including occlusions, chronic venous insufficiency and deep vein thrombosis. However, plethysmography devices are cumbersome and require a highly trained practitioner limiting their use. Plethysmography assessment also takes a considerable amount of time to complete, and typically lacks the potential to localize the site of stenosis/occlusion.

Doppler methods can provide information about the location of vascular dysfunction. Duplex ultrasound is the gold standard method for PVD diagnosis and is the most sensitive non-invasive method that currently exists. However, similar to plethysmographic techniques, expensive equipment and highly trained practitioners are required. Also, Doppler vascular assessment is even more time consuming than plethysmography. As such plethysmography and Doppler methods have limited use for PVD screening or everyday use in the clinic.

In contrast, ABI is relatively simple, fast and cheap and is recommended as an initial diagnostic tool in primary care, however it has poor sensitivity in patients with calcified arteries. Similar to plethysmography, ABI lacks localization capability. Other versions of brachial methods, such as the segmental blood pressure measurement, can assist pathology localization and toe brachial methods can counter medial artery calcification, increasing specificity for people with PAD and diabetes. However, segmental pressure measurement is not reliable and the Toe Brachial Index requires more time than an ABI, and specific environmental conditions.

Many noninvasive methods have been developed to aid the diagnosis of PVDs. However, given the rising number of patients with PVD and the shortcomings of current methods, there is still a need for new non-invasive diagnostic tools. The continuous effort for proposing noninvasive techniques for diagnosis of PVDs over more than 300 years further highlights the need for developing new diagnostic techniques. While Duplex ultrasound and plethysmography can be excellent means of PVD diagnosis, training requirements limit their more widespread use. Any potential alternative should be intuitive and simple to use, minimizing these training requirements. The high cost of Doppler ultrasound and plethysmography devices is another hurdle against their availability. While asymptomatic patients are also at high risk of morbidity/mortality, the time and cost of such diagnostic methods typically limits their use for symptomatic patients.

Ideally, a new solution will provide an early diagnosis of all three common types of PVD accurately, easily, cheaply, quickly and without extensive training. Advances in wearable technology and polymer-based sensors are probably the most likely candidates to address the clinician requirements for an ideal system. They may also provide continuous monitoring during functional activity and exercise, a capability that is notably missing in existing devices but which may add to diagnostic capability. No such wearable or polymer-based device is yet validated for diagnosis of any PVDs. However, the polymer-based technology is developing, with the prospect of providing early diagnosis due to their low-cost, minimum training requirement and the ability to provide relatively immediate information about both peripheral arterial and venous functionality.

## Additional file

**Additional file 1: Table S1.** Summary of the performance of the discussed noninvasive methods used for PVD diagnosis.

## Abbreviations

PVD: peripheral vascular disease
PAD: peripheral arterial disease
CVI: chronic venous insufficiency
DVT: deep vein thrombosis
RVF: residual volume fraction
SGP: strain-gauge plethysmography
APG: air plethysmography
PPG: photo plethysmography
LRR: light reflection rheography
IPG: impedance plethysmography
B-mode: brightness mode
CW: continuous wave
PW: pulsed wave
DU: duplex ultrasound
ABI: Ankle Brachial Index
TBI: Toe Brachial Index
PWV: pulse wave velocity
MRI: magnetic resonance imaging
VOTI: vascular optical tomographic imaging
ERB: electro-resistive band
HeMo: hemodynamic monitoring
